# Storytelling festival participation and tourists’ revisit intention

**DOI:** 10.3389/fpsyg.2022.968472

**Published:** 2022-09-07

**Authors:** Sung-Hoon Ko, Ji-Young Kim, Yongjun Choi, Jongsung Kim, Hyun Chul Kang

**Affiliations:** ^1^Graduate School of Education, Kyonggi University, Suwon, South Korea; ^2^Department of Business Administration, Hankuk University of Foreign Studies, Seoul, South Korea; ^3^College of Business Administration, Hongik University, Seoul, South Korea; ^4^College of General Education for Truth, Sincerity, and Love, Kyonggi University, Suwon, South Korea; ^5^College of Creative Engineering, Kyonggi University, Suwon, South Korea

**Keywords:** storytelling, local festival, authenticity, positive emotion, revisit intention

## Abstract

Storytelling is getting increasing attention as one of the effective strategies for revitalizing the local festivals and even regional economies. Yet, the mechanisms through how storytelling helps the success of local festivals are still relatively less known. Using the data from 322 individuals who participated in local festivals using storytelling, our results showed that local festival storytelling is positively related to tourists’ revisit intention. Furthermore, the positive relationship between local festival storytelling and tourists’ revisit intention was serially mediated by authenticity and positive emotion. Theoretical and practical implications are discussed.

## Introduction

Tourists experience positive emotions and become willing to revisit a region when a local festival meets their expectations; unmet expectations result in negative emotions, and tourists become less inclined to return to a region. Holding local festivals without differentiation reduces local economic profits and decreases tourist interest. Storytelling is emerging as a strategy to revitalize the local economy and build brand assets through its differentiation of festivals (Olsson et al., 2016).

Recently, COVID-19 has made the tourism industry one of the hardest hit sectors across the world. In this context, stimulating tourists’ positive emotions through storytelling can be more effective than other marketing strategies stimulating one’s reasons for traveling. Supporting this, the Danish futurist Rolf (1999) notes the ‘dream society’ in which the current society is changing from a rational-centered paradigm to an emotion-driven one. That is, he argues that our future will be dominated by dreams and emotions that are shaped by stories, thus, the emerging markets targeting one’s dream and emotions will surpass the existing markets based on information. More specifically, consumers experience pleasurable emotions through storytelling and share it with others through word of mouth (Delgadillo and Escalas, 2004; Holbrook, 2005). Therefore, storytelling is vital for the development and revitalization of the local tourism industry.

Local festivals that use storytelling contribute to the economy’s revitalization. For example, the ability of storytelling to promote regional growth for tourism development is highly recognized in the Netherlands (VROMraad, 2006). Local festivals using storytelling are also increasing in Korea. For example, as of 2017, the “Imsil N Cheese Festival” contributed KRW 2.3 billion in revenue through experiential tourism at Imsil Cheese Theme Park. The festival also added to sales of KRW 23.4 billion in dairy products such as cheese, having a significant impact on revitalizing the local economy. As such, tourists who have experienced the unique characteristics of a region through local festival storytelling perceive the local festival as simply a regional festival rather than a commercial endeavor and feel the authenticity of the local festival, which creates positive emotions. Therefore, it is essential to use storytelling that incorporates the unique characteristics and culture of a region to help build positive behavioral intentions to revisit and recommend.

This study explores the effects of local festival storytelling, especially on tourists’ revisit intentions. Specifically, this study reveals the mechanisms of how storytelling works, focusing on authenticity and positive emotions, showing that storytelling is an effective way to run a local festival and can be a major marketing tool for successful festival operation.

## Theoretical background and hypotheses development

Festivals are public ceremonies that convey a special meaning to visitors through various activities (Goldblatt, 2001). “Story” means something that occurred in the past, ‘telling’ emphasizes the present situation of the story, and finally “ing” means sharing the situation and interacting with it (Brown J. S. et al., 2005). Academically, storytelling is understood as a means and technique to integrate and utilize new media technologies in the creative process (Teehan, 2006). Memories are based on stories; the more contacts in a story, the more comparisons can be made with previous experiences, enabling increased learning (Schank, 1990). Through storytelling, the form of regional public relations is changing from existing methods of information delivery to the sharing of stories.

Storytelling emphasizes a local festival’s unique culture or characteristics, helping visitors to perceive its authenticity and uniqueness. Literature shows that the more consumers perceive a story provided by a specific brand as relevant to themselves, the more authentic they believe the brand is (Guber, 2007). Therefore, it can be predicted that visitors will perceive the authenticity of a festival through their experience of a positive bond with the region through local festival storytelling.

H1: Local festival storytelling will have a positive relationship with authenticity.

The authenticity of a local festival refers to its authentic representation of local traditions (Chhabra, 2005). In other words, it refers to the feeling that tourists perceive about the uniqueness of an area and its portrayal of regional “truths” and “facts” (Handler, 1986). Although tourists may be satisfied with the simulated images created by the media and pseudo-events (Boorstin, 1964), likely, they cannot experience the real authenticity of the tourist area through this.

Literature shows that when visitors’ perception of authenticity in historical and cultural sites is high, the overall satisfaction with tourist destinations increases (Naoi, 2004). Likewise, we predict that local festivals’ authenticity could effectively bring out visitors’ positive emotions. Mehrabian and Russell (1974) suggests that consumers’ emotions are triggered by stimuli. Supporting this, Price et al. (1995) demonstrated that service encounters’ authenticity play an important role in evoking consumers’ positive emotions. In a similar vein, Kim (2021) showed that, using the panel data in China, consumers who perceive high levels of service authenticity are more likely to experience positive emotions. Therefore, it can be predicted that there will be a positive relationship between local festivals’ authenticity and positive emotions.

H2: Local festivals’ authenticity will have a positive relationship with tourists’ positive emotions.

The intention to revisit a location is a key factor in predicting customer behavior. It leads to meaningful results in relationship marketing, as it refers to the will and belief that customers can determine their attitude or future behavior toward a specific object (Boulding et al., 1993). Revisit intention is influenced by various factors derived from a customer’s emotions, and a strong preference to revisit a location leads to positive behavior. Customers’ emotions can greatly affect their intention to return, as positive emotions related to consumption, such as excitement and pleasure, or negative emotions, such as boredom and anger, affect their consumption evaluation (Brown T. J. et al., 2005). The literature also shows that the revisit intention decreases for customers who feel negative emotions but increases for customers who experience positive emotions (Oliver, 1981). Thus, this study sets the following hypothesis:

H3: Positive emotion toward local festivals will have a positive relationship with tourists’ revisit intention.

Storytelling can strengthen the meaning of the existing physical environment and reinforce the region’s unique identity by finding hidden histories and forming a story based on cultural background or history (Saarinen, 2006). It can elicit the participation of tourists, arouse interest in the local area, and change behavior (Moscardo, 1996; Van Dijk, 2011). The literature also confirms that storytelling can positively affect tourists’ word of mouth and visit intentions (Akgün et al., 2015). Therefore, this study establishes the following hypothesis.

H4: Local festival storytelling will have a positive relationship with tourists’ revisit intention.

Since human memory is based on stories (Schank, 1990), local festival storytelling can positively impact tourists and future visitors. Similarly, unlike existing advertisements that emphasize only factual aspects, storytelling used in advertising tries to convey brand value based on emotional stories (Weick, 1995).

Local festival storytelling can fundamentally convey local values to tourists and elicit a persuasive effect through emotional stories. Therefore, participants in a local festival that uses storytelling will perceive the festival as more authentic, and as a result, will experience positive emotions. This authenticity orients toward consumer value rather than commercial motive (Beverland et al., 2008). Naoi (2004) further demonstrated a positive relationship between authenticity and satisfaction, a positive emotion.

As tourists experience local festival storytelling, their perceived authenticity is an important factor influencing their future positive emotion building, directly related to behavioral intentions. In particular, the Affect Control Theory (ACT) posits that people act according to the emotions formed by an event that reaffirms what they feel (Heise, 1989). Therefore, it can be inferred that positive feelings derived from the perception of authenticity will affect the revisiting behavior of tourists. Thus, this study establishes the following hypothesis on the double-mediated effect of authenticity and positive emotion on the relationship between local festival storytelling and tourist behavioral intentions.

H5: The relationship between local festival storytelling and tourist behavioral intentions will be double mediated by authenticity and positive emotion about local festivals.

## Materials and methods

### Study participants and procedure

Surveys were administered to visitors to the Jeju Wildfire Festival, a local festival featuring storytelling. Jeju Island is one of the most loved destinations in South Korea. Among many local festivals in Jeju Island, we chose the Jeju Wildfire Festival because it includes the three elements (i.e., story, expression, and interactions) of storytelling (Tilden, 2009). During this festival, the researcher distributed and collected questionnaires from festival participants. A total of 350 questionnaires were collected. Eighteen questionnaires with a centralization tendency or incomplete answers were excluded; thus, 332 were entered into statistical data analysis. The respondents consisted of 132 men (39.8%) and 200 women (60.2%); by age, 65 respondents were in their 20s (19.6%), 78 in their 30s (23.5%), 56 in their 40s (16.9%), 79 in their 50s (23.8%), and 54 in their 60s (16.3%). For the participation motivation, 65 respondents marked “by chance” (19.6%), 216 “to spend leisure time” (65.1%), 12 “because it was an event held nearby” (3.6%), and 4 “because the festival has been designated by the Ministry of Culture, Sports and Tourism as the best festival” (1.2%). For the sources of information about the festival, 112 respondents marked “TV/Radio” (33.7%), 10 “the Internet (festival-related website)” (3.0%), 6 “the Internet (news, blog, Mape, etc.)” (1.8%), 46 “SNS (Facebook, Twitter)” (13.9%), 6 “street posters and leaflets (promotional brochures)” (1.8%), 146 “close friends” (44.0%), and 6 “others” (1.8%).

### Measures

Respondents were asked to answer the survey questions on a 5-point Likert scale ranging from 1 “not at all” to 5 “strongly agree.” Because all the measures we used were originally written in English, we followed Brislin (1970) back-translation method to translate the original items into Korean. Specifically, the whole process was done by an academic fluent in both languages.

#### Local festival storytelling

Drawing on Tilden (2009) and Yavuz et al. (2016), the construct of local festival storytelling is composed of three sub-dimensions: story, expression, and interaction. Question items were adapted to the context of local festival storytelling in South Korea. The items included “the story of this festival contains regional characteristics” to measure the sub-dimension of the story, “experience events express the feeling of the festival’s story” to measure expression, and “the story and the way of expression of this festival made me feel attracted to it” to measure interaction. The Cronbach’s α was 0.910.

#### The authenticity of local festivals

The measurement items used in Price et al. (1995) were adapted to measure the construct of authenticity in this study. The question items included “I perceived authenticity about the actions of the festival organizers when I participated in the main festival” and “I perceived humanity in the actions of the festival organizers when I participated in the main festival.” The Cronbach’s α was 0.951.

#### Positive emotions

The question items developed and used by Lilius et al. (2008) were adapted to the context of this study to measure the construct of positive emotion. The items include “I feel joy when I participate in this festival” and “I feel very positive emotions when I participate in this festival program.” Cronbach’s α was 0.917.

#### Revisit intention

The items used in Conner and Sparks (1996) and Oliver (1997) were modified to the context of this study to measure the construct of revisit intention. The items include “I am willing to revisit this festival” and “I will revisit this festival even if it costs extra money.” Cronbach’s α was 0.935.

## Results

### Confirmatory factor analysis

The goodness of fit of the research model in this study was determined through Confirmatory Factor Analysis (CFA), and the results are as follows: χ^2^(248) = 1281.209 (*p* = 0.000), CFI = 0.910, TLI = 0.882, NFI = 0.901, IFI = 0.910, RMSEA = 0.052, and RMR = 0.019, indicating that the overall goodness of fit exceeds the fit criteria. Next, the internal consistency of latent variables was determined with the coefficient of Cronbach’s α; all coefficients were greater than 0.9; thus, we concluded we have good internal consistency. Furthermore, the Average Variance Extracted (AVE) value of 0.6 or higher indicates that the research model satisfies the traditional criteria.

### Hypotheses testing

Table 1 presents the construct means, standard deviations, and correlations. Correlations among our study variables ranged from –0.383 to 0.448, As expected, local festival storytelling was positively related to authenticity (*r* = 0.448, *p* < 0.01) and revisit intention (*r* = 0.344, *p* < 0.01). In addition, authenticity was positively related to positive emotions (*r* = 0.315, *p* < 0.01), and positive emotions was positively related to revisit intention (*r* = 0.383, *p* < 0.01).

**TABLE 1:** Construct means, standard deviations, and correlations.

	Mean	SD	1	2	3	4
1. Local festival Storytelling	3.864	0.321	*0.815*			
2. Authenticity	3.468	0.551	0.448**	*0.861*		
3. Positive emotions	3.684	0.493	0.412**	0.315**	*0.813*	
4. Revisit intention	3.892	0.332	0.344**	0.254**	0.383**	*0.775*

**p < 0.01. The italic numbers in the diagonal are the square root of the AVE.

AMOS 24.0 was used to test the hypothesis of this study, and the test results are as follows. Table 2 summarizes the structural equation modeling results. The path coefficient between local festival storytelling and authenticity was β = 0.767 (*t* = 9.110, *p* < 0.001), supporting Hypothesis 1. The path coefficient between local festival storytelling and positive emotion was β = 0.282 (*t* = 6.031, *p* < 0.001), supporting Hypothesis 2. The path coefficient between positive emotion and revisit intention was β = 0.232 (*t* = 4.491, *p* < 0.001), supporting Hypothesis 3. Finally, the path coefficient between local festival storytelling and revisit intention was β = 0.195 (*t* = 5.809, *p* < 0.05), supporting Hypothesis 4.

**TABLE 2:** Path analysis results.

H	Path	b	SE	CR	*p*
H1	Local festival storytelling → Authenticity	0.767	0.084	9.110	<0.001
H2	Authenticity → Positive emotions	0.282	0.047	6.031	<0.001
H3	Positive emotions → Tourist’s behavioral intention	0.232	0.052	4.491	<0.001
H4	Local festival storytelling → Revisit intention	0.195	0.034	5.809	<0.001

To test Hypothesis 5, a serial mediation hypothesis, we used bootstrapping (Preacher and Hayes, 2004, 2008), and the results are presented in Table 3. The indirect effect of local festival storytelling on revisit intention via authenticity and positive emotion was positive and significant (effect = 0.021; LLCI_95_ = 0.004, ULCI_95_ = 0.043) as the confidence interval does not include zero. Thus, Hypothesis 5 was supported.

**TABLE 3:** Indirect effects of the double-mediation impacts (authenticity and positive emotions).

	Effect	LLCI95%	ULCI95%	BootSE
Total indirect effect	0.168	0.113	0.230	0.029
Local festival storytelling → Authenticity → Revisit intention	0.122	0.071	0.182	0.028
Local festival storytelling → Positive emotions → Revisit intention	0.024	0.005	0.053	0.012
Local festival storytelling → Authenticity → Positive emotions Revisit intention	0.021	0.004	0.043	0.009

### Common methods bias

Because we used self-reported data to test our hypotheses, common method bias issues cannot be completely ruled out. Thus, by controlling for the latent variable factors, we tested if our results could be biased by our research design (Podsakoff et al., 2003).

The results of analyzing common methods bias are presented in Table 4. The model fit indices were χ^2^(248) = 1281.209, *p* < 0.001, CFI = 0.910, TLI = 0.882, IFI = 0.910, RMSEA = 0.052, and RMR = 0.019 before controlling for the effects of a latent methods factor and χ^2^(222) = 1051.021, *p* < 0.001, CFI = 0.928, TLI = 0.894, IFI = 0.928, RMSEA = 0.049, and RMR = 0.017 after controlling for it. As a result of the comparison, the difference in Δ value (230.188, *p* > 0.05) according to the difference in degrees of freedom (df = 26) between the two models was found to be insignificant, suggesting that the probability of occurrence of the same method bias in this model is low. In addition, the absolute value of λ-CMV, which is the difference between the λ values before and after controlling, never exceeded 0.2. Thus, we concluded that the common method bias is not a serious issue in our research model.

**TABLE 4:** Analysis of common method bias.

	χ^ 2^	df	*p*	χ^2^/df	RMSEA	CFI	IFI	TLI
Measurement Model (M.M)	1281.209	248	<0.001	5.166	0.052	0.910	0.910	0.882
Controlled Model (C.M)	1051.021	222	<0.001	4.734	0.049	0.928	0.928	0.894
Stepwise χ^2^ Analysis	△ χ^ 2^	△df		Accepted Model				
M.M-C.M	230.188	26	>0.05	Measurement Model				

## Discussion

### Theoretical implications

The theoretical implications of this study are as follows. First, this study revealed a causal relationship between local festivals that utilize storytelling and the authenticity that tourists feel about local festivals. Particularly, the study provides a theoretical implication by examining the reaction of tourists through applying the storytelling technique, a brand strategy (Ryu et al., 2018; Kao, 2019; Hong et al., 2021) for products or brands, to local festivals in the tourism industry.

Second, this study empirically demonstrated the dual mediating effect of authenticity and positive emotions in the relationship between local festival storytelling and revisit intention. Whereas most previous studies on storytelling have focused on either its antecedents (e.g., McCabe and Foster, 2006; Zhong et al., 2017) or outcomes (Bassano et al., 2019), our results showed the psychological mechanism though how the storytelling at the festival could influence the tourists’ revisit intention providing more fruitful ways of advancing our knowledge on the roles of storytelling in festivals.

Third, the construct of local festival storytelling was not unidimensional in this study. Rather, it was composed and measured in three dimensions and then meaningfully applied to this study to show how local festival storytelling leads to tourists’ intentions to revisit based on authenticity and positive emotions toward the festival. In addition, given that tourists’ emotional experience could be important in intangible culture heritage as well as heritage tourism (Butler et al., 2003; Pérez-Gálvez et al., 2019), this study demonstrates the importance of storytelling in local festivals for stimulating tourists’ positive emotional experiences.

Lastly, this study contributes to the literature by showing how storytelling can be utilized as a strategy to differentiate local festivals to revitalize the local economy. Storytelling is an active differentiation strategy for determining the success of a local festival that is carried out to revive the local economy. Storytelling is a critical element to consider when hosting a local festival as it can increase tourists’ behavioral intentions to revisit the region.

### Practical implications

First, our findings show that it is necessary to pay attention to storytelling as a strategic plan for a region holding local festivals or making regional revitalization plans, suggesting that it can be instrumental in increasing tourists’ revisit intentions to these areas. Local festivals without differentiation cannot elicit the sympathy of tourists as it is difficult to regard a festival without storytelling as a genuine activity to promote a region; rather, it may cause adverse effects on local festivals, resulting in a negative local image. Therefore, places that wish to hold local festivals need to consider this cautiously so tourists can perceive these festivals as authentic and meaningful activities.

Second, it is beneficial for a region to elicit tourists’ positive emotions and behavioral intentions. Local festival visitors build positive or negative emotions through their experiences. When positive emotions are formed, it can lead to the positive behavior of tourists, but when negative emotions are formed, derogatory word of mouth may spread, and intention to revisit sharply decreases. Therefore, it is crucial to consider storytelling as a strategy that can create advantages such as authenticity and positive emotions in tourists. In other words, tourists who have experienced local festival storytelling build positive emotions according to the authenticity of the local festival, thereby increasing revisit intentions, suggesting that storytelling can be effective in forming local brand assets through local festivals. More specifically, from the tourism management perspective, they may need to take an experience-centric approach by providing tourists with a positive tourism experience via the appropriate use of storytelling (Ritchie et al., 2011; Bec et al., 2019; Cervera-Taulet et al., 2019; Oshriyeh and Capriello, 2022) which drive them to revisit the regions in the future.

### Limitations

While the results of this study can be considered significant, the study has the following limitations. First, the generalization of the findings in this study to the storytelling of various local festivals may be limited because the survey was conducted with tourists at only one local festival. Since each region has its own unique characteristics and culture, it may be difficult to apply the findings from this study to all local festivals.

Second, studies on the elements constituting local festival storytelling are still insufficient, and theories have not been universally established. Therefore, it may be difficult to generalize the elements constituting local festival storytelling used in this study to other studies because the various unique factors that make up local festival storytelling were not reflected in this study.

## Conclusion

This study examined the relationships among local festival storytelling, authenticity, positive emotions, and revisit intention. Our empirical results showed the serial mediating effect of perceived authenticity and positive emotions in the positive relationship between local festival storytelling and tourists’ revisit intention demonstrating the usefulness of storytelling in revitalizing local tourism industry. By demonstrating the psychological paths through how local festival storytelling could bring positive impacts in local economy, our results provide fruitful ways forward on the studies on local festivals and local tourism industry. Moreover, given the economic hardship in the tourism sector due to the COVID-19 pandemic, our results provide managerial insights about how to make the festivals more successful and even revitalize the local tourism industry.

## Data availability statement

The raw data supporting the conclusions of this article will be made available by the authors, without undue reservation.

## Ethics statement

Ethical review and approval was not required for the study on human participants in accordance with the local legislation and institutional requirements. The patients/participants provided their written informed consent to participate in this study.

## Author contributions

S-HK and J-YK contributed to the conception and design of the study. S-HK performed the statistical analysis. YC contributed to the writing of the original manuscript and supplemented several parts of this manuscript. JK and HK wrote the sections of the manuscript. All authors contributed to manuscript revision, read, and approved the submitted version.
